# Characterization of pulsations in the brain and cerebrospinal fluid using ultra-high field magnetic resonance imaging

**DOI:** 10.3389/fnins.2024.1305939

**Published:** 2024-05-09

**Authors:** Tiago Martins, Bruno de Almeida, Minjie Wu, Kristine A. Wilckens, Davneet Minhas, James W. Ibinson, Howard J. Aizenstein, Tales Santini, Tamer S. Ibrahim

**Affiliations:** ^1^Department of Bioengineering, University of Pittsburgh, Pittsburgh, PA, United States; ^2^Department of Psychiatry, University of Pittsburgh, Pittsburgh, PA, United States; ^3^Department of Radiology, University of Pittsburgh, Pittsburgh, PA, United States; ^4^Department of Anesthesiology and Perioperative Medicine, University of Pittsburgh, Pittsburgh, PA, United States

**Keywords:** cerebrospinal fluid (CSF), magnetic resonance imaging (MRI), ultra-high field (7 Tesla), brain clearance, echo-planar imaging (EPI), physiological brain pulsations

## Abstract

The development of innovative non-invasive neuroimaging methods and biomarkers is critical for studying brain disease. Imaging of cerebrospinal fluid (CSF) pulsatility may inform the brain fluid dynamics involved in clearance of cerebral metabolic waste. In this work, we developed a methodology to characterize the frequency and spatial localization of whole brain CSF pulsations in humans. Using 7 Tesla (T) human magnetic resonance imaging (MRI) and ultrafast echo-planar imaging (EPI), *in-vivo* images were obtained to capture pulsations of the CSF signal. Physiological data were simultaneously collected and compared with the 7 T MR data. The primary components of signal pulsations were identified using spectral analysis, with the most evident frequency bands identified around 0.3, 1.2, and 2.4 Hz. These pulsations were mapped spatially and temporally onto the MR image domain and temporally onto the physiological measures of electrocardiogram and respiration. We identified peaks in CSF pulsations that were distinct from peaks in grey matter and white matter regions. This methodology may provide novel *in vivo* biomarkers of disrupted brain fluid dynamics.

## 1 Introduction

Clearance and exchange of brain fluids promote brain health by removing neurotoxic metabolic byproducts from the brain such as amyloid beta and tau (Nedergaard, 2013; Xie et al., 2013). Clearance of brain fluids is driven by pulsations of the perivascular spaces from autonomic nervous system (ANS) activity, which may vary as a function of brain states such as sleep and wakefulness (Herculano-Houzel, 2013; Xie et al., 2013; Hauglund et al., 2020) as well as brain diseases such as Alzheimer’s disease (Ramanathan et al., 2015; Peng et al., 2016) and major depressive disorder (Hock et al., 1998; Pomara et al., 2012).

Concurrently, the glymphatic system facilitates the convection of cerebrospinal fluid (CSF) between the peri-arterial and peri-venous spaces. This convective flow is thought to at least partially be driven by cardiac-induced blood flow pulsations along the arteries (Adolph et al., 1967; Schroth and Klose, 1992; Martin et al., 2012; Iliff et al., 2013). Although the specific mechanisms of clearance remain under debate, there is evidence that water is propelled by the arterial pulsations through aquaporin channels and supports solute transport from extracellular interstitial spaces, through perivascular spaces, and into CSF spaces. CSF and waste products from the brain are then pushed from parenchyma to subarachnoid spaces and may eventually be cleared via arachnoid granulations and dural and nasal lymphatic vessels (Rennels et al., 1990; Iliff et al., 2013; Kiviniemi et al., 2016; de Leon et al., 2017), or as recently discovered, the meningeal lymphatic vessels (mLVs) (Louveau et al., 2015; Ahn et al., 2019).

Development of quantitative CSF imaging methods is critical to understand factors that influence the CSF dynamics and brain fluid clearance. T1-weighted magnetic resonance imaging (MRI) with intrathecal injection of a gadolinium (Gd)-based contrast agent has been used to characterize CSF flow in human participants with idiopathic normal pressure hydrocephalus (iNPH) and dementia (Ringstad et al., 2017, 2018; Eide and Ringstad, 2019). This technique has afforded fully quantitative, high-resolution imaging of CSF and interstitial fluid (ISF) flow throughout the whole head but is highly invasive, as it requires a lumbar puncture. Gd may also remain deposited in the brain, limiting its longitudinal research utility (Gulani et al., 2017).

Fast acquisition functional magnetic resonance imaging (fMRI) paradigms have also been used to characterize CSF dynamics in iNPH and Alzheimer’s disease (AD) patients and in healthy control participants during sleep (Fultz et al., 2019; Shanks et al., 2019; Yamada et al., 2020; Yang et al., 2022). A resting-state fMRI study has evidenced a potential coupling between CSF pulsations and the global blood oxygen level-dependent (BOLD), which is notably reduced in patients with AD-related diseases, where the CSF region of interest (ROI) was considered at the bottom slice of the acquisitions and delineated using masks obtained from T2*-weighted fMRI and further confirmed with T1-weighted images (Han et al., 2021). While non-invasive, these sequences have relatively poor signal-to-noise ratio (SNR) and spatial resolution and have been limited to narrow fields of view encompassing only the 4th ventricular, bottom edge slice of the acquisitions or cerebral aqueduct. Moreover, similar fMRI techniques were employed comparing CSF pulsations in different regions such as edge slices and 4th ventricle, revealing distinct behaviors depending on the region selected for assessing the CSF dynamics (Kim et al., 2022).

The bulk changes in blood volume at the capillary level could cause widespread fluctuations of measured signal intensity with the cardiac cycle. Furthermore, large vessel pulsatility may cause tissue and CSF movement and production of an influx of unsaturated blood into the slice of interest affecting the measured signal intensity in the areas adjacent to the vessels. This leads to a signal variation when using echo-planar imaging (EPI) acquisitions (Dagli et al., 1999). Hence, fMRI and other techniques have been used to characterize different sources of pulsations in the brain (Poncelet et al., 1992; Biswal et al., 1995; Purdon and Weisskoff, 1998; Dagli et al., 1999; Kiviniemi et al., 2000). Thus, using MRI of CSF dynamics can inform the study of brain diseases and the role of sleep–wake states (Xie et al., 2013; Fultz et al., 2019).

Based on 3 Tesla (T) MRI magnetic resonance encephalography (MREG), it has been demonstrated that AD patients experience abnormalities in cardiovascular brain impulses, which can manifest as slow, fast, or even in a reverse direction of propagation (Rajna et al., 2021). Similarly, patients with epilepsy (Kananen et al., 2018, 2020, 2022) and narcolepsy (Järvelä et al., 2022) also have exhibited altered physiological brain pulsations. MREG imaging have also been used to identify changes in physiological brain pulsations during nonrapid eye movement (NREM) sleep (Helakari et al., 2022), and to identify the spatial location of brain physiological pulsations as well as the multiple sources of BOLD signal (Raitamaa et al., 2021). Despite being a non-invasive technique with high temporal resolution used to characterize alterations in brain pulsations dynamics based on fMRI measurements, MREG’s limited accessibility stands in contrast to the more broadly available fast EPI methods.

Alternative techniques for ultrafast EPI acquisitions have been proposed. These include inverse imaging (InI) (Lin et al., 2012), generalized inverse imaging (GIN) (Boyacioglu et al., 2013), and multi-slab echo-volumar imaging (multi-slab EVI or MEVI) (Posse et al., 2012, 2013). These techniques offer a fast-sampling rate and reduced sensitivity to physiological noise. However, they come with the trade-off of potential loss of spatial resolution or introduction of geometrical distortions (Lin et al., 2012; Posse et al., 2012, 2013; Boyacioglu et al., 2013). Moreover, these studies have primarily utilized 3T MRI scanners.

Ultra-high field MRI (≥ 7 T) provides a major advantage of increased SNR, which can be used either to increase the resolution of the images or to decrease the scanning time (with the use of higher acceleration factors). The 7 T field strength also has higher sensitivity to BOLD signal and better vasculature conspicuity (Moser et al., 2012; Santini et al., 2021b).

Using ultrafast EPI we acquired the CSF MR signal in real-time. We report CSF pulsation patterns through spectral analysis. We applied the same methodology across datasets of seven different participants to validate the observed results. To identify whether spectral peaks in CSF pulsatility aligned with changes in physiological measures thought to drive CSF flow, we simultaneously collected physiological measures of electrocardiogram (ECG) and respiration in one participant.

## 2 Methods

The overall design of this study is based on two main steps: (1) *In-vivo* 7 T image acquisition, concurrent with physiology measurements, and (2) image processing with spectral analysis. The processing and analysis of the power-frequency spectrum and its corresponding spatial mappings were fine tuned for detection of bandwidth and peak span, thresholding levels for masks, and smoothing degrees for filtering.

Participants provided informed consent as approved by the University of Pittsburgh’s Institutional Review Board (identification number PRO17030036). Seven healthy volunteers (two male, age range 26–30 years old and five female, age range 21–25 years old) were scanned to obtain EPI data. From one volunteer, we obtained two axial and one sagittal slices. Whole brain EPI data, including the cerebellum, were collected from five participants. For the last participant, we collected simultaneous EPI and physiological data.

### 2.1 Image acquisition

Images were acquired using a whole-body 7 T MRI system (Siemens 7 T MAGNETOM) and with the human-connectome EPI multiband MR sequence, which can be obtained from https://www.cmrr.umn.edu/multiband/index.shtml (Moeller et al., 2010; Uğurbil et al., 2013). The sequence obtains fast acquisitions, high signal contrast of the CSF pulsation, and high sensitivity to BOLD signal, thus being well-suited for studies of sleep and neurodegenerative and psychological disorders. The imaging was acquired with an in-house developed and fabricated head coil with a 16-channel Tic-Tac-Toe transmit array with a 32-channel receive insert (Santini et al., 2018; Krishnamurthy et al., 2019; Santini et al., 2021b) that is load insensitive (Ibrahim et al., 2008; Kim et al., 2016; Santini et al., 2021b) and capable of whole brain homogenous imaging at 7 T (Ibrahim et al., 2013). By using this coil design, we were able to acquire signal from the entire brain with minimal regions of significant excitation losses and using the single transmit mode of the 7 T scanner.

The acquired EPI images yield a real-time visualization of the brain pulsations. The sequence was optimized to perform fast brain imaging. For Volunteer 1, during the development of the protocol, the EPI acquisition was broken into two sequences: a 2-slice axial view, with echo time (TE) of 17 ms, repetition time (TR) of 102 ms, acceleration factor of 3, field of view (FOV) of 220 mm × 220 mm, resolution 1.5 mm × 1.5 mm × 3 mm, total acquisition time of 1 min and 8 s; and a 1-slice sagittal view, with TE of 18 ms, TR of 100 ms, acceleration factor of 3, field of view (FOV) of 216 mm × 216 mm, resolution 1.5 mm × 1.5 mm × 4.4 mm, total acquisition time of 1 min and 3 s. In both cases, a total of 600 volumes were sequentially acquired.

For five volunteers (Volunteers 2 to 6), whole-brain imaging was acquired. The main data acquisition was done with TE of 20 ms, TR of 155 ms, isotropic resolution of 2 mm, and acceleration factor of 2. The FOV was 192 mm × 192 mm. The acquisition was broken into 19 slabs of 3 axial slices each for a total of 57 slices, where each slice has a thickness of 2 mm, totaling 114 mm, providing a whole-brain coverage. A total of 600 volumes were sequentially acquired per slab in a single sequence run for an acquisition time of 1 min and 36 s per slab. For Volunteer 2, the EPI acquisition was done using TR of 152 ms but only 15 slabs of 3 axial slices for a total of 45 axial slices. Another EPI acquisition on the same volunteer was also performed using TR of 51 ms and a single slice.

In all cases, the TE values were chosen for future potential BOLD analysis. Furthermore, all EPI images were acquired from volunteers in a resting state condition. Moreover, spin-echo EPIs were also acquired for B_0_ field distortion correction with the same phase encoding (PE) direction of the EPI acquisition (PA) and with the opposite PE direction (AP). The sequence parameters included a TE of 39.4 ms, TR of 6,000 ms, with the other parameters – such as field of view, resolution, number of slices, echo spacing, and position - matched to those of the EPI sequence.

A T1-weighted imaging (MPRAGE) sequence was used for proper localization of the EPI field of view and as a structural scan for the image processing. This acquisition was done using 0.75 mm isotropic resolution, TR of 3,000 ms, TE of 2.17 ms, and 256 slices for a coverage of 240 mm x 173 mm x 192 mm in total time of acquisition of ~5 min.

### 2.2 Physiological measurements

Electrocardiogram (ECG) and respiration signals were collected for one participant (Volunteer 7) inside the MR scanner using MR compatible ECG leads and an expansion belt attached to the chest to track inflation and deflation of the chest during respiration. Acquisition was digitalized using BIOPAC system (ECG: Cardiology | Research | BIOPAC, n.d.; Respiration Transducer for MRI | TSD221-MRI | Research | BIOPAC, n.d.). The simultaneously collected data allowed temporal signal analysis of both MR and physiologic signals. The imaging data acquired in conjunction with the physiological data used a TR of 75 ms, TE of 28 ms, and 3 axial slices (4 mm-thick each) including the lateral ventricles.

### 2.3 Image processing

The processing pipeline was developed based on MATLAB (MATLAB - MathWorks, n.d.), ANTs (Avants et al., 2009), and FSL (Jenkinson et al., 2012) packages. It consisted of denoising, distortion correction, bias correction, and skull stripping of each dataset volume. The initial step was loading the slabs and merging them into a single dataset (necessary only for whole-brain coverage images). Next, denoising was performed using a noise estimation tool with variance stabilization transformation (VST) for Rician-distributed noise (Foi, 2011). The Rician heteroscedastic noise was converted to a homoscedastic noise after the forward VST. The block-matching 4D (BM4D) denoising algorithm (Maggioni et al., 2013) could then be applied and the denoised image is obtained after the inverse VST. This tool has been used for other MRI applications (Santini et al., 2021a) and yields a good result when applied to EPI data. Distortion correction was performed using the estimated B_0_ maps derived from the spin-echo sequence using the *topup* tool (Andersson et al., 2003) from FSL software package. The generated map was used for correction of the EPI data. Then, the images were bias corrected using the N4 (Tustison et al., 2010) tool from the ANTs software package with spline distance parameter of 200. The final skull stripping was performed using the FSL brain extraction tool (BET). We note the merging of images captured at different times, as each slab is acquired within distinct time frames. However, the transition of the analysis to the frequency domain, with the fast Fourier transform (FFT) executed for each voxel, mitigated these temporal differences. For the subsequent spectral analysis, the whole-brain coverage images were segmented using SynthSeg (Billot et al., 2023a,b), allowing the assessment of the brain pulsations in three different regions: cortical gray matter (cGM), cerebral white matter (cWM) and CSF.

### 2.4 Spectral analysis

The frequency analysis was performed for each dataset individually and resulted in both a frequency power spectrum and a mask for brain localization of specific frequency bands. A frequency spectrum was calculated for arbitrarily selected points for validation of the findings across different brain regions, for single slices (performed for images not covering the entire brain), and also for the regions described in the previous section (whole-brain, cGM, cWM and CSF).

After processing each individual EPI data, the frequency processing and analysis were performed in MATLAB and Python. The time series of each voxel was used to generate a frequency spectrum using FFT. With the 600 volumes of 152/155 ms TR acquisition, the frequency resolution of the frequency spectrum is 0.011 Hz and the maximum frequency is 3.289/3.226 Hz. The same frequency analysis for the 51 ms TR data produces a much larger frequency spectrum of up to 9.8 Hz. Therefore, frequency components higher than 3 Hz can be observed and analyzed. The analysis was done using both individual points manually selected as well as the average across the 2D or 3D space. For acquisitions encompassing a 2D/3D region of interest (ROI), the FFT was calculated for each voxel, and subsequently the spectral intensity values across all voxels were averaged for each frequency, resulting in a single intensity value per frequency for each 2D/3D ROI.

#### 2.4.1 Spectral peaks identification

**Figure 1:**
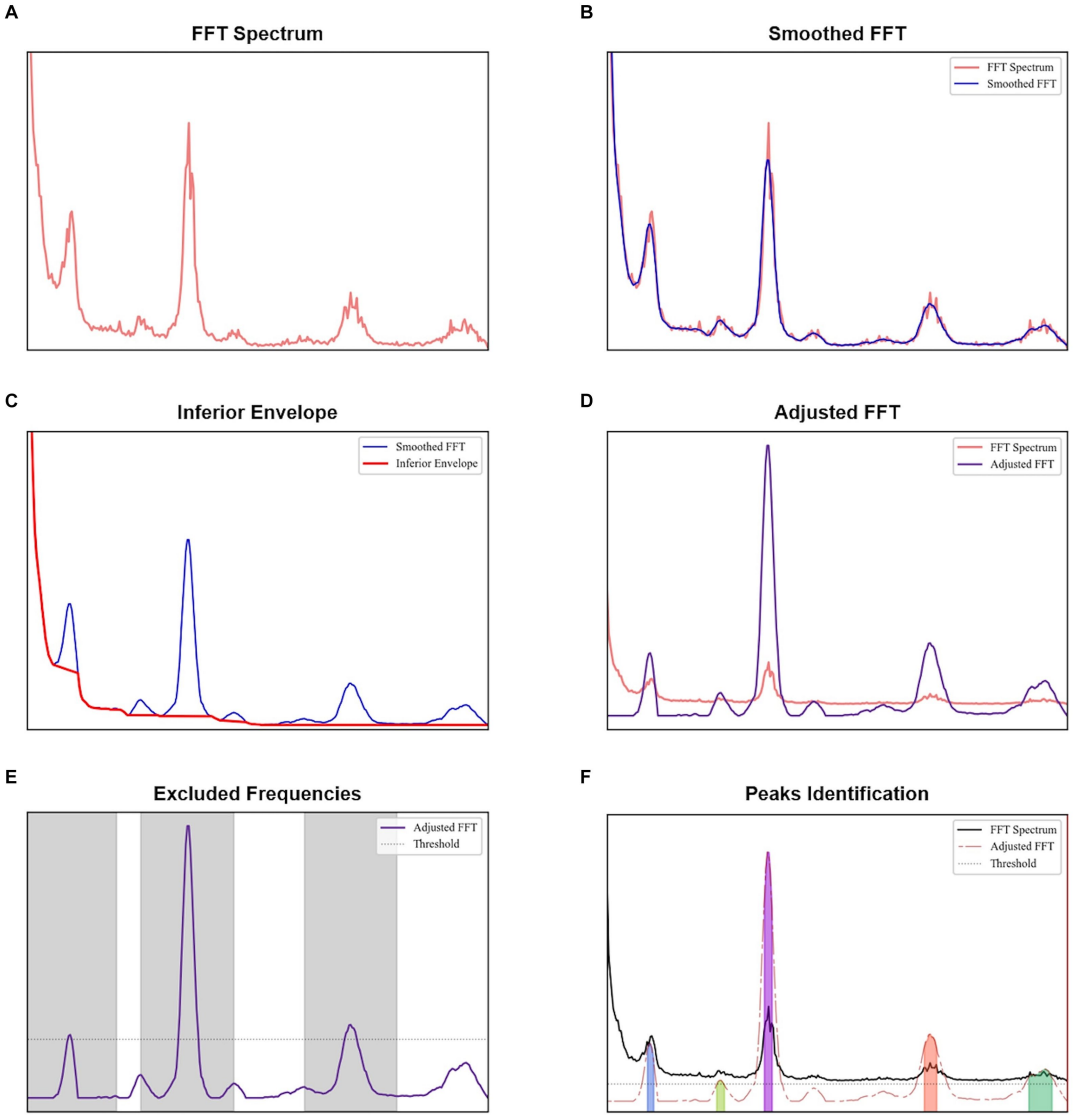
Procedure to identify peaks for each spectral analysis: **(A)** original FFT obtained, **(B)** smoothed FFT after the application of Savitzky–Golay filter, **(C)** obtaining the inferior envelope, **(D)** adjusted FFT obtained after dividing each value of the smoothed FFT by the inferior envelope, **(E)** identification of the most evident peaks, with a height higher than a threshold defined as the sum of the mean and standard deviation of the adjusted FFT, and **(F)** peaks identified in the adjusted FFT with a minimum height given by the sum of the mean and standard deviation of the regions not excluded in the previous plot.

To identify the relevant peaks of each spectrum, an algorithm was developed in Python as depicted in Figure 1. Initially, each spectrum plot was smoothed using a Savitzky–Golay filter of order 2. The filter window was defined as 0.133 Hz, corresponding to the normal range of 12 to 20 breaths per minute in adults (Sapra et al., 2020). For a 51 ms TR, the number of samples of this window was doubled.

The lower envelope observed across all spectra, which is a characteristic of the acquisition as evidenced by tests with a phantom (provided in the Supplementary material), was modeled by identifying and accumulating the minimum values along the frequency axis. Whenever the observed values exceed these minimums, indicating an increase in the signal, linear interpolation was employed to bridge between the identified minimum value and the subsequent minimum value observed when the signal decreased again. The smoothed spectrum was then divided by this envelope to reflect the relative increase. By subtracting one unit from this normalized spectrum, we obtained an adjusted spectrum optimized for peak identification.

To establish a baseline level, the most evident peaks in each adjusted spectrum were identified first. We calculated the mean and standard deviation of the adjusted spectrum, and their sum was used as the minimum threshold for detecting primary peaks (*findpeaks* implementation in Python). An exclusion zone of 0.667 Hz was applied around each identified peak, where this frequency range corresponds to the normal cardiac range of 60 to 100 beats per minute in healthy adults (Sapra et al., 2020). Regions in the adjusted spectrum outside the exclusion zones were assessed to establish a baseline level.

A spectrum peak was identified as those with a minimum height equal to the sum of the mean and standard deviation of the baseline level. To prevent small oscillations above this height from being classified as peaks, a minimum prominence of 1.5 dB was required.

#### 2.4.2 Spectral spatial analysis

Spatial analysis was done by creating image masks based on the localization of voxels with significant signal in each frequency band. It was also performed for single-slice acquisitions, covering the 2D space acquired. Moreover, due to whole-brain coverage, a similar approach was employed for the entire brain images and specific brain regions (cGM, cWM and CSF).

Regarding the voxel analysis, the power map of a given frequency band was determined voxel-wise by averaging respective power values within the frequency band. For better visualization, each power map was then binarized with a chosen threshold (75% of the peak amplitude of the corresponding frequency band) and spatially smoothed using a Gaussian filter (sigma of 1.6), generating the final masks for each frequency band. These masks were overlaid on the original EPI and T1 weighted acquisitions for anatomical reference. The T1 weighted image was registered with the average EPI image of the dataset using SPM12.

For single-slice, whole-brain and brain regions (cGM, cWM and CSF), peaks were identified as shown in the previous section. For each of these peaks, the bandwidth corresponding to a 3 dB drop ($\sqrt{2}/2$ of the peak magnitude) and the area under the spectrum within this bandwidth were determined, aiming to identify potential biomarkers.

## 3 Results

**Figure 2:**
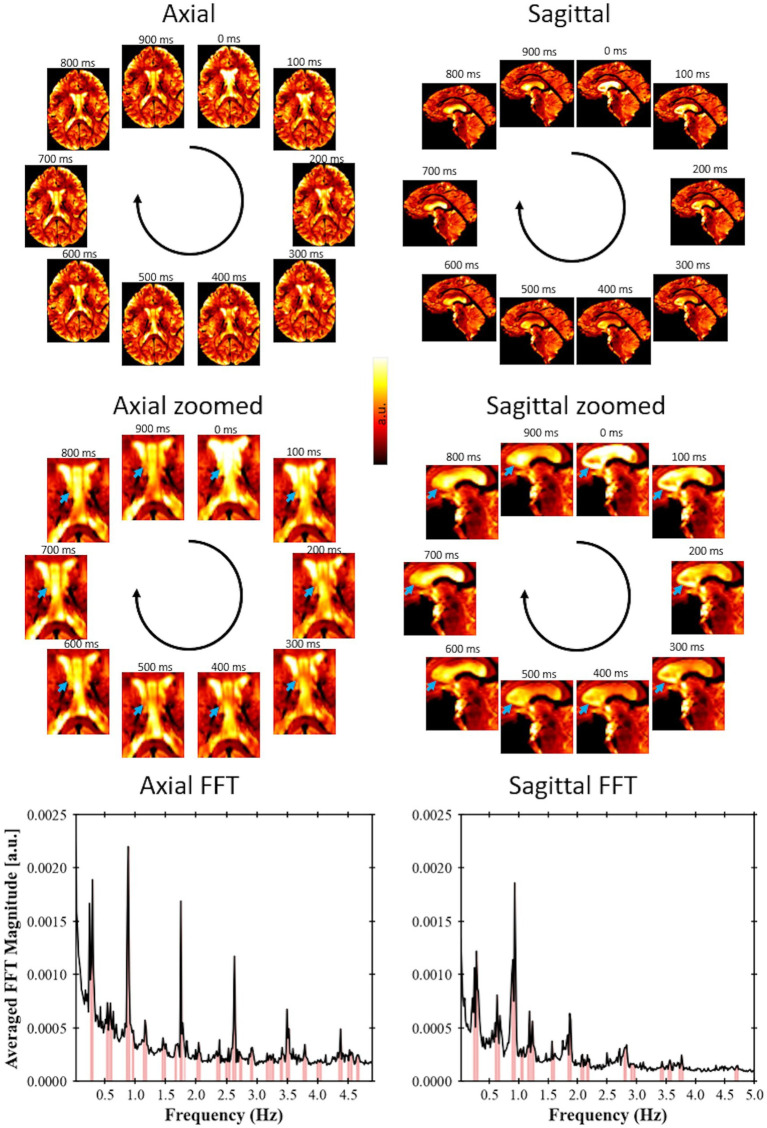
Fast EPI acquisition (TR = 102 ms for axial view, TR = 100 ms for sagittal view) for Volunteer 1 showing signal changes due to CSF flow; axial slices with a spatial resolution of 1.5 × 1.5 × 3 mm and a sagittal slice with spatial resolution of 1.5 × 1.5 × 4.4 mm. The blue arrows point to regions of large variation in signal over time. A video showing these pulsations in real time is available at doi: 10.6084/m9.figshare.24022932. For each view, a single-slice FFT spectrum is shown, where the peaks were identified following the procedure proposed in this study.

A video was created based on the image series of the fast EPI data after acquisition and processing in Volunteer 1 (Figure 2). The video visually indicates the presence of periodical signal from the CSF flow. For this first volunteer, the acquired image covers only a few slices. However, it was still possible to apply the developed methodology to identify relevant frequencies, demonstrating the potential of using this approach even with single-slice acquisitions.

**Figure 3:**
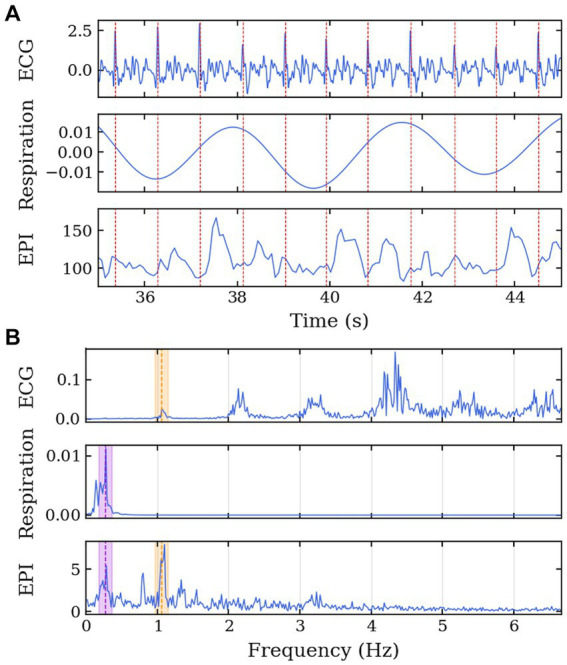
MRI data acquisition along the cardiac cycle and respiration for Volunteer 7. Echo-planar imaging acquisition performed with concurrent physiological measurement of electrocardiogram in the 7 T scanner. **(A)** Time series of ECG, respiration belt, and EPI signals. The EPI signal was temporally aligned with the physiological data using an external trigger signal from the scanner. The red lines represent the R-peaks of the ECG. **(B)** Frequency spectrum of the ECG, respiration belt, and EPI signals. The purple region highlights the common frequency band between the respiration and EPI signals, and the orange region highlights the common frequency band between the ECG and EPI signals.

To confirm the presence of physiological signals such as respiration and cardiac motion, CSF temporal data was aligned with measurements from the electrocardiogram and respiration belt in Volunteer 7 for visual comparison of similarity between the physiological activities and the change in signal intensity from CSF regions (Figure 3A). The frequency spectrum of the datasets was also aligned following the same comparison as the time series data (Figure 3B). The two major signal bands were highlighted between the ECG and CSF data (around 1.1 Hz) and the respiration belt and CSF data (around 0.3 Hz).

**Figure 4:**
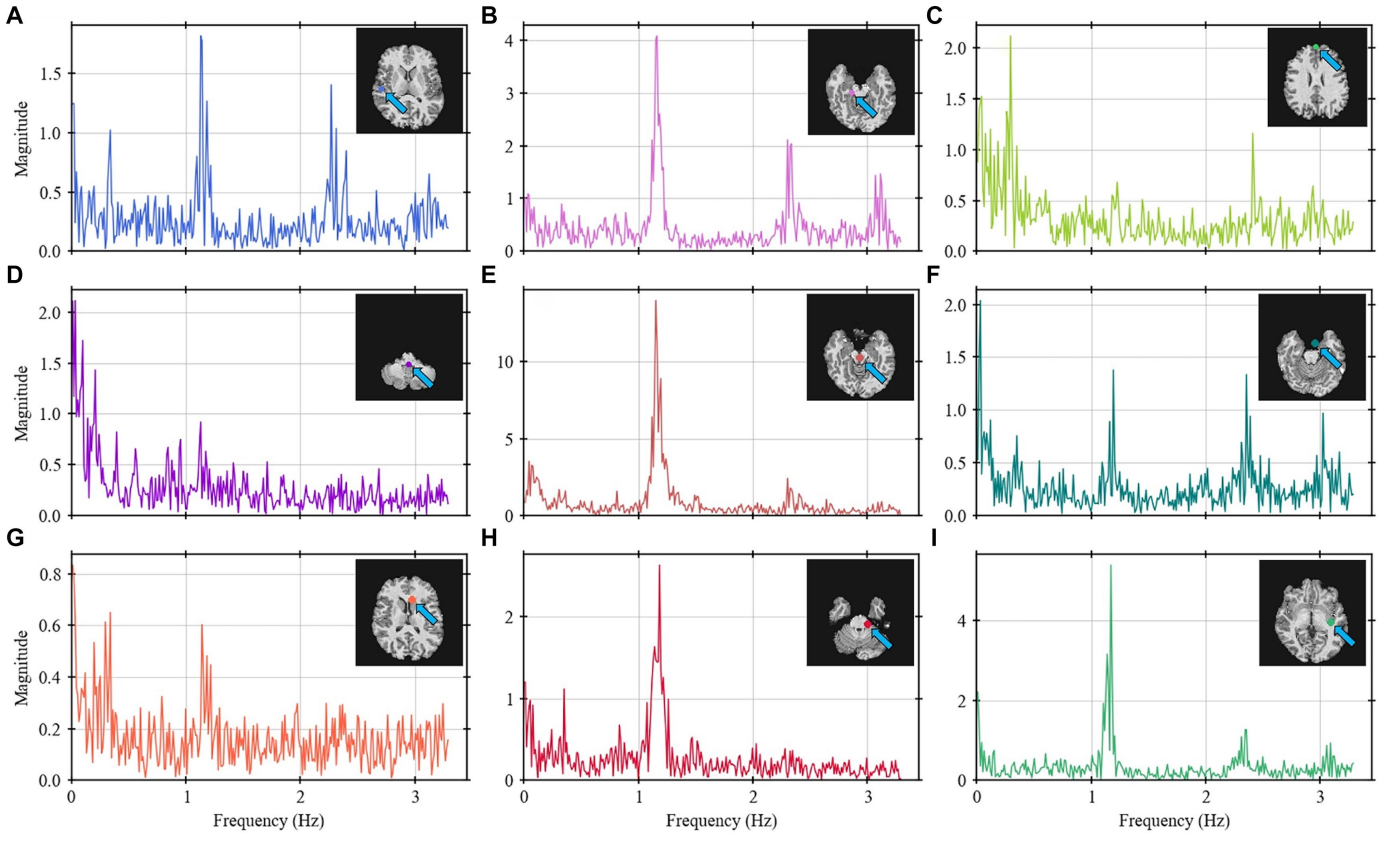
Frequency spectrum for nine selected points **(A–I)** throughout the brain for Volunteer 2. Some points show higher intensity on the 1.2 and 2.4 Hz bands (points **A,B,E,F,G–I**) whereas other show more on the 0.3 Hz band (points **C,D,G**). The labels describe the brain anatomy where the data was obtained.

To verify that various points of the brain contribute differently on the frequency spectrum, Figure 4 represents the frequency spectrum for 9 arbitrary points throughout the brain. The position of each point is described by the brain anatomy that it belongs to as shown on the top-right corner of each spectrum graph. Most of the points show frequencies around 1.2 Hz. Depending on the position, the signal shows the 0.3 Hz and/or the 2.4 Hz bands.

**Figure 5:**
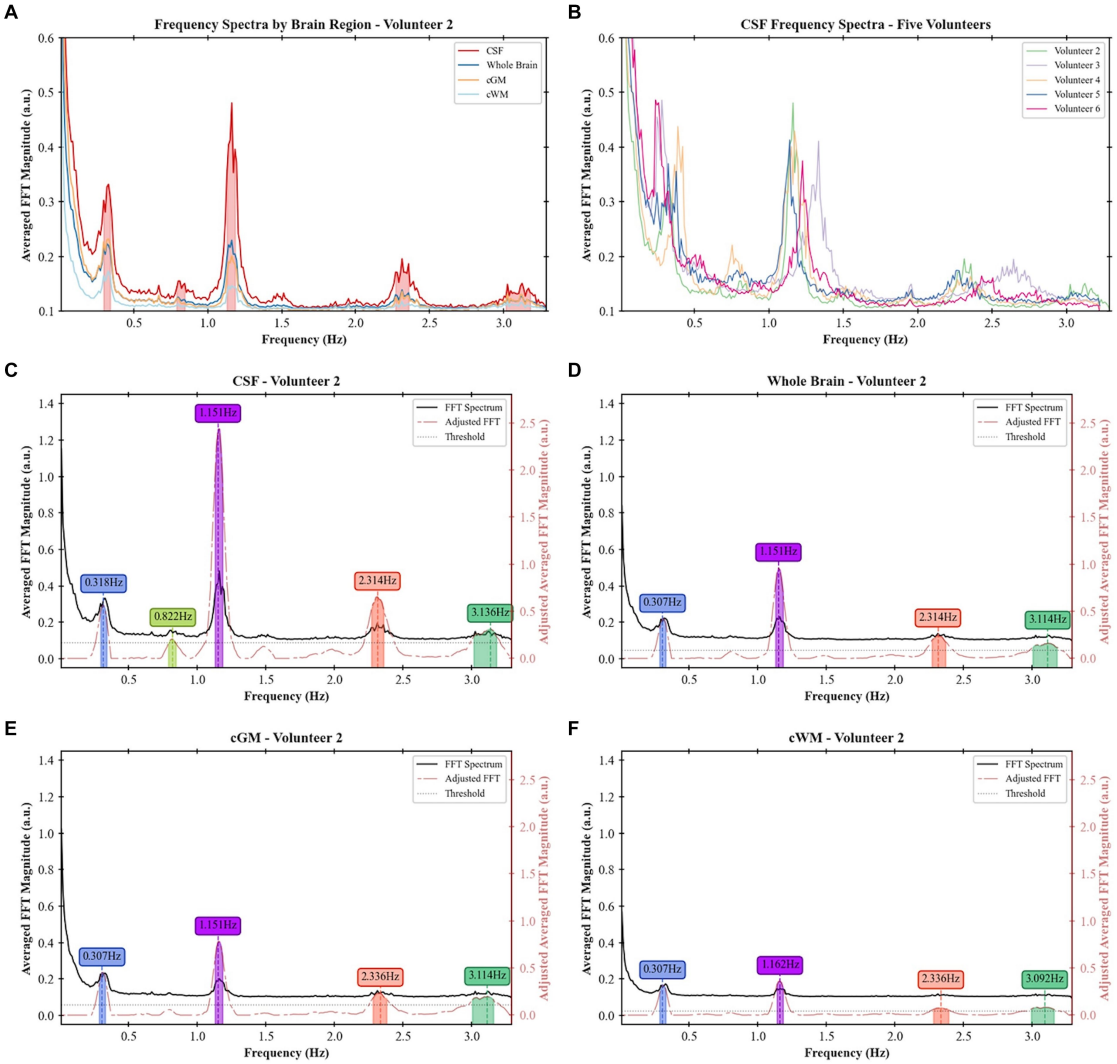
Frequency spectra and identified peaks in different participants and brain regions: **(A)** frequency spectra for the whole brain, cGM, cWM, and CSF regions for Volunteer 2, **(B)** frequency spectra of CSF across five different volunteers, **(C)** frequency bands identified in CSF regions for Volunteer 2, **(D)** frequency bands identified in the whole-brain coverage for Volunteer 2, **(E)** frequency bands identified in cGM for Volunteer 2, and **(F)** frequency bands identified in cWM for Volunteer 2.

A frequency analysis was obtained from the EPI data for the single-slices acquisitions, whole-brain coverage, and in the following brain regions: cGM, cWM and CSF. Based on a developed methodology to identify relevant peaks (Figure 1), the most significant frequency bands were highlighted, and for each of them a peak magnitude, bandwidth for a 3 dB drop and area under this range were obtained (Figure 5). For all volunteers with whole-brain coverage, bands with similar center frequencies of approximately 0.3, 1.2, and 2.4 Hz can be identified. Table 1 shows the center frequency for the bands calculated for each volunteer. The frequency bands with centers at 0.3 and 1.2 Hz closely approximate the respiration and cardiac frequencies of a human adult (around 18 breaths per minute and 72 heart beats per minute, respectively). These bands can be identified as Band 1 and Band 4 on Table 1. In terms of magnitudes discovered within these frequency bands for CSF regions, their averages are 0.594 ± 0.378 (a.u.) and 1.747 ± 0.475 (a.u.), respectively. For the areas, their averages are 0.027 ± 0.017 (Hz) and 0.119 ± 0.037 (Hz), respectively. Finally, for the bandwidths, their averages are 0.053 ± 0.006 (Hz) and 0.078 ± 0.016 (Hz), respectively.

**Table 1:** Values of identified frequency bands and respective magnitudes, areas under the curve and bandwidths for 3 dB drop in whole-brain coverage imaging.

		**Band 1 (0.247–0.387 Hz)**				**Band 2 (0.484–0.516 Hz)**				**Band 3 (0.763–0.914 Hz)**				**Band 4 (1.129–1.312 Hz)**			
		**Frequency (Hz)**	**Magnitude (a.u.)**	**Area (Hz)**	**Bandwidth (Hz)**	**Frequency (Hz)**	**Magnitude (a.u.)**	**Area (Hz)**	**Bandwidth (Hz)**	**Frequency (Hz)**	**Magnitude (a.u.)**	**Area (Hz)**	**Bandwidth (Hz)**	**Frequency (Hz)**	**Magnitude (a.u.)**	**Area (Hz)**	**Bandwidth (Hz)**
**CSF**	**Volunteer 2**	0.318	0.565	0.026	0.056	–	–	–	–	0.822	0.206	0.012	0.065	1.151	2.435	0.142	0.066
	**Volunteer 3**	0.269	0.597	0.028	0.056	0.516	0.082	0.003	0.043	–	–	–	–	1.312	1.507	0.115	0.085
	**Volunteer 4**	0.387	1.197	0.054	0.057	–	–	–	–	0.763	0.496	0.039	0.080	1.151	2.048	0.169	0.103
	**Volunteer 5**	0.333	0.162	0.006	0.043	–	–	–	–	0.806	0.119	0.006	0.072	1.129	1.374	0.095	0.068
	**Volunteer 6**	0.247	0.450	0.021	0.053	0.484	0.073	0.005	0.070	0.914	0.128	0.008	0.074	1.226	1.369	0.075	0.068
	**Mean**	0.311	0.594	0.027	0.053	0.500	0.077	0.004	0.057	0.826	0.237	0.016	0.073	1.194	1.747	0.119	0.078
	**Std Dev**	0.055	0.378	0.017	0.006	0.023	0.006	0.001	0.019	0.064	0.177	0.015	0.006	0.076	0.475	0.037	0.016
**Whole Brain**	**Volunteer 2**	0.307	0.421	0.020	0.057	–	–	–	–	–	–	–	–	1.151	0.961	0.056	0.065
	**Volunteer 3**	0.269	0.477	0.022	0.055	–	–	–	–	–	–	–	–	1.301	0.828	0.067	0.103
	**Volunteer 4**	0.387	0.769	0.035	0.056	–	–	–	–	0.763	0.208	0.016	0.084	1.151	0.977	0.094	0.107
	**Volunteer 5**	0.333	0.070	0.002	0.028	–	–	–	–	0.806	0.051	0.003	0.066	1.129	0.840	0.058	0.069
	**Volunteer 6**	0.247	0.354	0.016	0.054	0.516	0.050	0.002	0.057	0.914	0.045	0.003	0.066	1.226	0.715	0.039	0.068
	**Mean**	0.309	0.418	0.019	0.050	0.516	0.050	0.002	0.057	0.828	0.101	0.007	0.072	1.192	0.864	0.063	0.082
	**Std Dev**	0.055	0.251	0.012	0.012	–	–	–	–	0.078	0.092	0.008	0.010	0.071	0.107	0.020	0.021
**cGM**	**Volunteer 2**	0.307	0.453	0.021	0.055	–	–	–	–	–	–	–	–	1.151	0.781	0.046	0.065
	**Volunteer 3**	0.269	0.510	0.024	0.054	–	–	–	–	–	–	–	–	1.301	0.735	0.059	0.076
	**Volunteer 4**	0.387	0.647	0.031	0.052	–	–	–	–	0.774	0.177	0.016	0.088	1.161	0.882	0.066	0.089
	**Volunteer 5**	0.344	0.077	0.002	0.035	–	–	–	–	–	–	–	–	1.129	0.824	0.045	0.067
	**Volunteer 6**	0.247	0.315	0.011	0.050	0.516	0.082	0.004	0.057	–	–	–	–	1.226	0.711	0.040	0.066
	**Mean**	0.311	0.401	0.018	0.049	0.516	0.082	0.004	0.057	0.774	0.177	0.016	0.088	1.194	0.786	0.051	0.073
	**Std Dev**	0.056	0.216	0.011	0.008	–	–	–	–	–	–	–	–	0.070	0.069	0.011	0.010
**cWM**	**Volunteer 2**	0.307	0.315	0.015	0.055	–	–	–	–	–	–	–	–	1.162	0.363	0.016	0.061
	**Volunteer 3**	0.269	0.382	0.018	0.054	–	–	–	–	–	–	–	–	1.301	0.253	0.018	0.090
	**Volunteer 4**	0.387	0.423	0.025	0.056	–	–	–	–	0.774	0.067	0.004	0.069	1.161	0.376	0.030	0.081
	**Volunteer 5**	0.344	0.157	0.006	0.045	–	–	–	–	–	–	–	–	1.140	0.501	0.028	0.063
	**Volunteer 6**	0.247	0.230	0.011	0.055	–	–		–	–	–	–	–	1.226	0.391	0.022	0.067
	**Mean**	0.311	0.302	0.015	0.053	–	–	–	–	0.774	0.067	0.004	0.069	1.198	0.377	0.023	0.072
	**Std Dev**	0.056	0.109	0.007	0.005	–	–	–	–	–	–	–	–	0.066	0.088	0.006	0.013

		**Band 5 (1.935 to 1.946 Hz)**				**Band 6 (2.247 to 2.452 Hz)**				**Band 7 (2.602 to 2.774 Hz)**				**Band 8 (2.914 to 3.136 Hz)**			
		**Frequency (Hz)**	**Magnitude (a.u.)**	**Area (Hz)**	**Bandwidth (Hz)**	**Frequency (Hz)**	**Magnitude (a.u.)**	**Area (Hz)**	**Bandwidth (Hz)**	**Frequency (Hz)**	**Magnitude (a.u.)**	**Area (Hz)**	**Bandwidth (Hz)**	**Frequency (Hz)**	**Peak Magnitude (a.u.)**	**Area (Hz)**	**Bandwidth (Hz)**
**CSF**	**Volunteer 2**	–	–	–	–	2.314	0.655	0.054	0.107	–	–	–	–	3.136	0.315	0.047	0.176
	**Volunteer 3**	1.946	0.143	0.007	0.051	–	–	–	–	2.613	0.480	0.077	0.202	–	–	–	–
	**Volunteer 4**	–	–	–	–	2.355	0.355	0.057	0.180	–	–	–	–	–	–	–	–
	**Volunteer 5**	1.935	0.110	0.008	0.069	2.247	0.415	0.036	0.094	–	–	–	–	3.054	0.133	0.014	0.119
	**Volunteer 6**	–	–	–	–	2.452	0.448	0.047	0.118	2.774	0.211	0.036	0.175	–	–	–	–
	**Mean**	1.941	0.126	0.007	0.060	2.342	0.468	0.049	0.125	2.694	0.346	0.057	0.189	3.095	0.224	0.031	0.148
	**Std Dev**	0.008	0.023	0.001	0.013	0.086	0.131	0.009	0.038	0.114	0.190	0.029	0.019	0.058	0.129	0.024	0.040
**Whole Brain**	**Volunteer 2**	–	–	–	–	2.314	0.241	0.023	0.114	–	–	–	–	3.114	0.161	0.026	0.188
	**Volunteer 3**	–	–	–	–	–	–	–	–	2.613	0.253	0.038	0.171	–	–	–	–
	**Volunteer 4**	–	–	–	–	2.355	0.146	0.025	0.197	–	–	–	–	–	–	–	–
	**Volunteer 5**	1.935	0.071	0.004	0.065	2.247	0.244	0.026	0.116	–	–	–	–	3.065	0.080	0.008	0.124
	**Volunteer 6**	1.935	0.047	0.003	0.056	2.452	0.218	0.022	0.124	2.774	0.118	0.018	0.174	–	–	–	–
	**Mean**	1.935	0.059	0.003	0.061	2.342	0.212	0.024	0.138	2.694	0.186	0.028	0.173	3.090	0.121	0.017	0.156
	**Std Dev**	0.000	0.017	0.001	0.006	0.086	0.046	0.001	0.040	0.114	0.095	0.014	0.002	0.035	0.058	0.013	0.045
**cGM**	**Volunteer 2**	–	–	–	–	2.336	0.236	0.023	0.109	–	–	–	–	3.114	0.198	0.028	0.174
	**Volunteer 3**	1.946	0.107	0.005	0.053	–	–	–	–	2.613	0.241	0.032	0.154	–	–	–	–
	**Volunteer 4**	–	–	–	–	2.355	0.158	0.019	0.155	–	–	–	–	–	–	–	–
	**Volunteer 5**	1.935	0.083	0.004	0.062	2.247	0.242	0.031	0.152	–	–	–	–	3.075	0.111	0.010	0.117
	**Volunteer 6**	1.935	0.054	0.003	0.049	2.452	0.235	0.025	0.117	2.774	0.149	0.021	0.167	–	–	–	–
	**Mean**	1.939	0.081	0.004	0.055	2.348	0.218	0.025	0.133	2.694	0.195	0.026	0.161	3.095	0.155	0.019	0.146
	**Std Dev**	0.006	0.027	0.001	0.007	0.084	0.040	0.005	0.024	0.114	0.065	0.008	0.009	0.028	0.062	0.013	0.040
**cWM**	**Volunteer 2**	–	–	–	–	2.336	0.080	0.008	0.120	–	–	–	–	3.092	0.086	0.014	0.171
	**Volunteer 3**	1.935	0.044	0.002	0.054	–	–	–	–	2.602	0.069	0.008	0.141	–	–	–	–
	**Volunteer 4**	–	–	–	–	2.355	0.054	0.005	0.097	–	–	–	–	–	–	–	–
	**Volunteer 5**	1.946	0.049	0.003	0.074	2.323	0.138	0.020	0.159	–	–	–	–	3.097	0.067	0.007	0.125
	**Volunteer 6**	1.935	0.027	0.002	0.057	2.441	0.110	0.009	0.098	2.774	0.068	0.009	0.151	2.914	0.035	0.002	0.073
	**Mean**	1.939	0.040	0.002	0.062	2.364	0.096	0.011	0.119	2.688	0.068	0.009	0.146	3.034	0.063	0.007	0.123
	**Std Dev**	0.006	0.012	0.001	0.011	0.053	0.037	0.007	0.029	0.122	0.001	0.000	0.007	0.104	0.026	0.006	0.049

**Figure 6:**
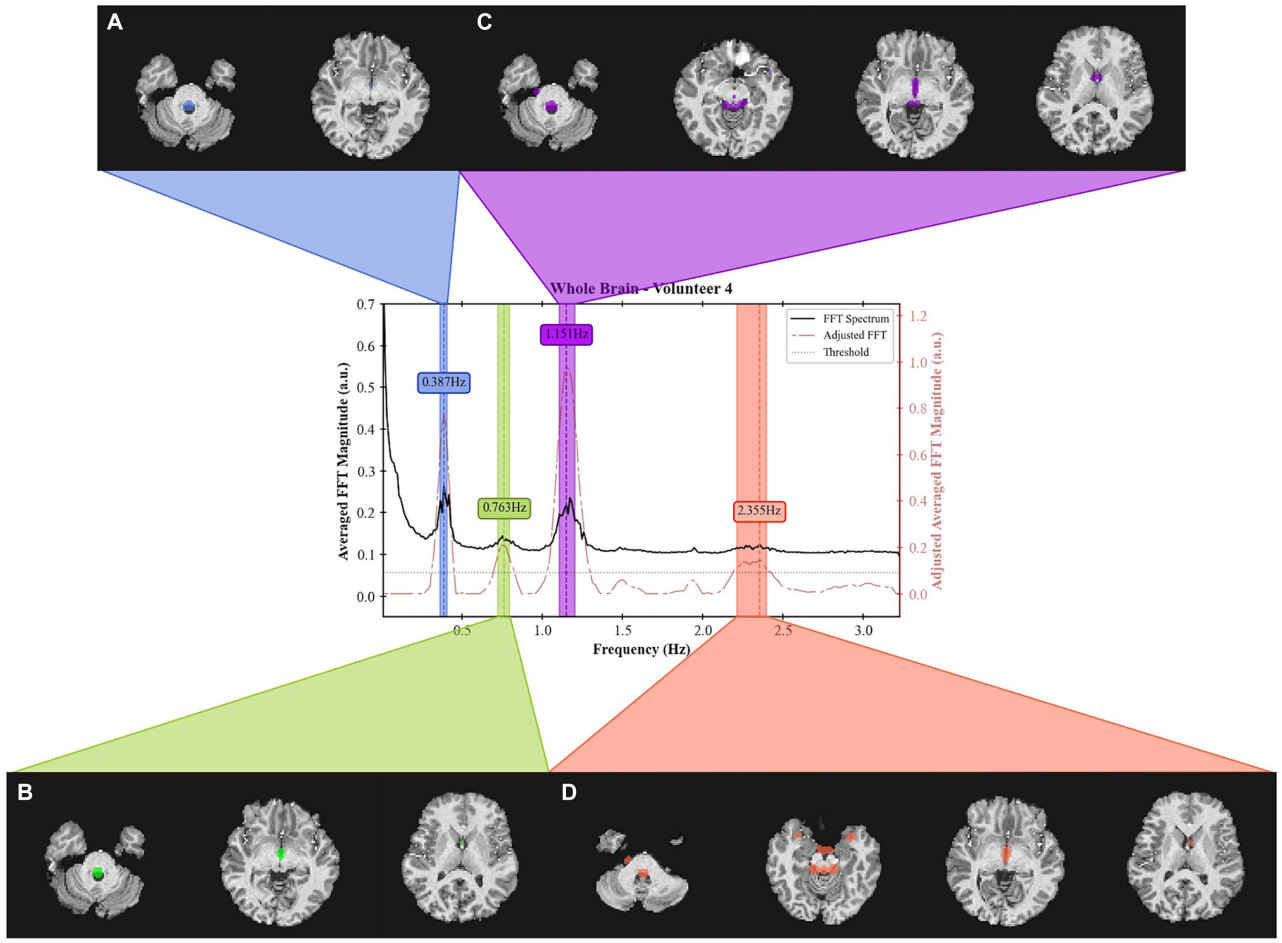
Frequency spectrum of Volunteer 4 with spatial localization of the signal from 4 separate frequency bands. For each band, a spatial mask was applied to the T1 weighted image. The acquired data can be obtained from the inferior region of the brain (cerebellum) up to the middle of the brain. The bandwidth for each band is defined as those that result in a 3 dB drop. The center frequencies are **(A)** 0.387 Hz, **(B)** 0.763 Hz, **(C)** 1.151 Hz, and **(D)** 2.355 Hz. The acquisition was done using an EPI sequence with TR = 155 ms with 19 slabs of 3 slices each for a total of 57 slices.

**Figure 7:**
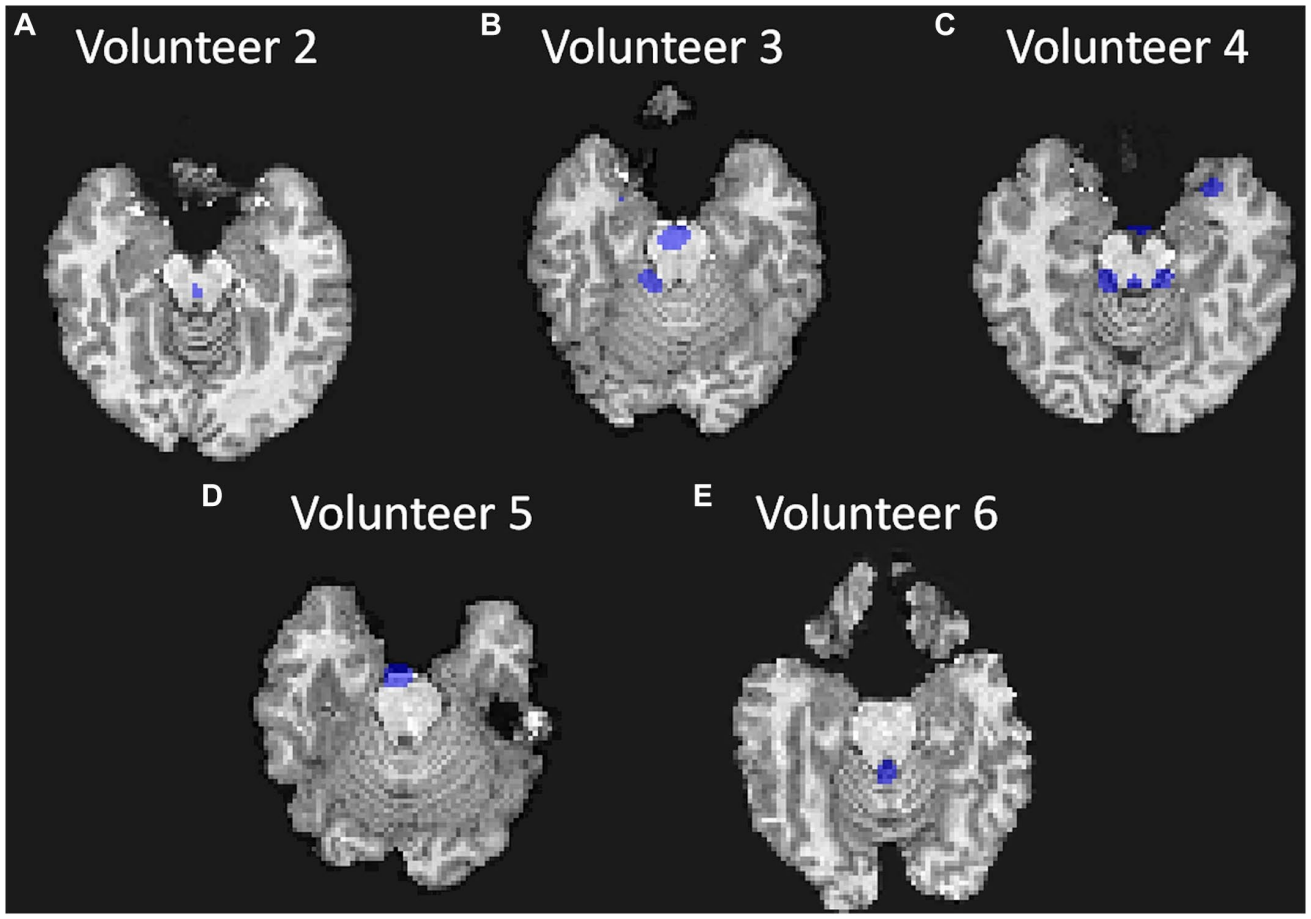
Visualization of the mask created for each volunteer at approximately the same position in the brain (bottom of the brain and top of the cerebellum and at approximately the same frequency band); **(A)** for Volunteer 2 at 1.151 Hz; **(B)** for Volunteer 3 at 1.301 Hz; **(C)** for Volunteer 4 at 1.151 Hz; **(D)** for Volunteer 5 at 1.129 Hz; and **(E)** for Volunteer 6 at 1.226 Hz.

The masks created per frequency band (Figure 6) show a spatial localization for the frequency band centered at lower frequencies, e.g., 0.387 Hz (Figure 6A) and 1.151 Hz (Figure 6C) overlapping with brain regions with larger volume of CSF (the main ventricles and cerebral aqueduct). Similar patterns were observed for all volunteers as shown in Figure 7 as the mask for the heart rate band is demonstrated in each of the volunteer’s data.

**Figure 8:**
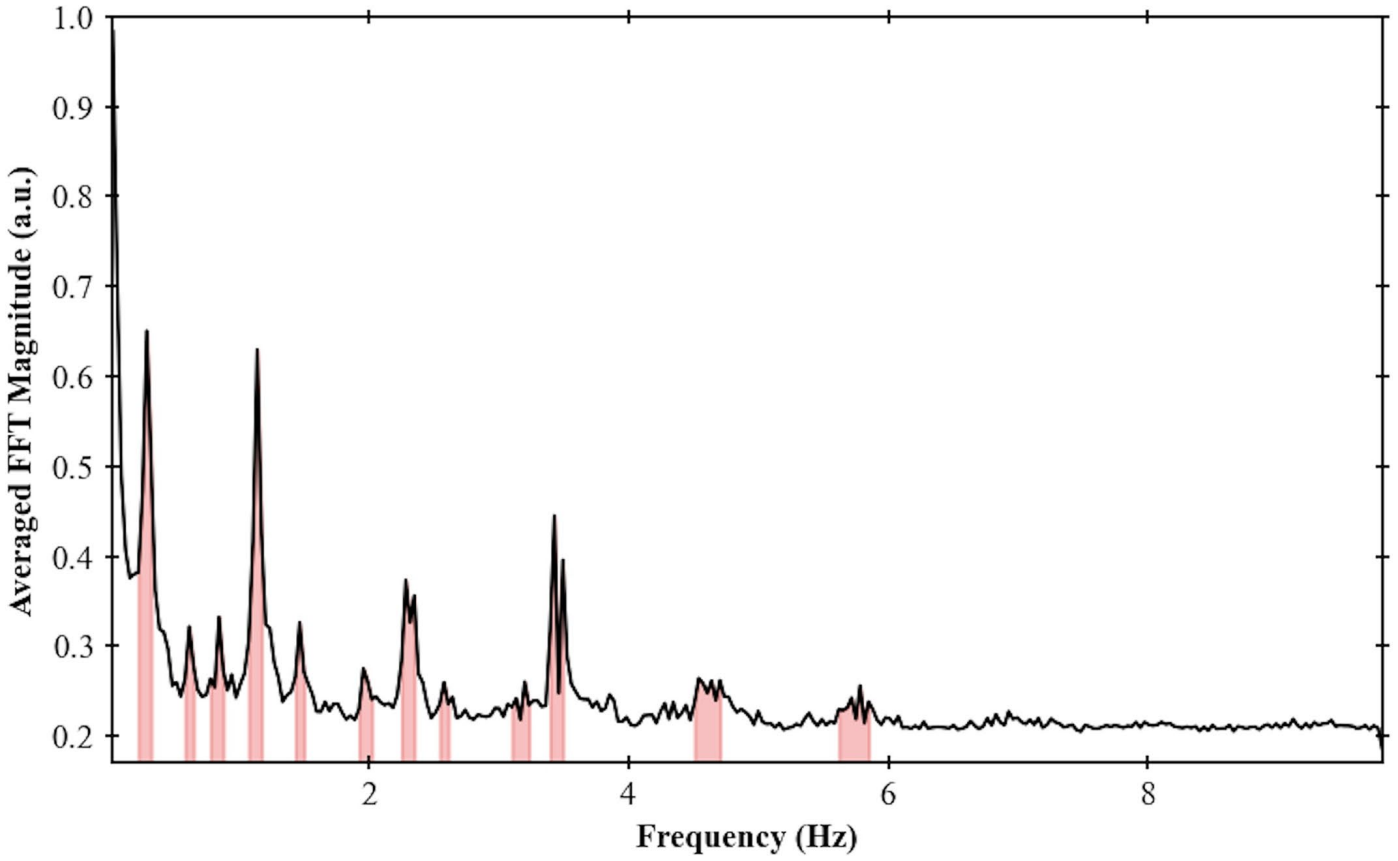
Frequency spectrum for Volunteer 2 done using an EPI sequence single slice with TR = 51 ms. Maximum frequency of 9.8 Hz and frequency bands were highlighted based on the procedure developed in this work.

For the larger frequency spectrum (dataset with TR of 51 ms), extra bands can be identified, and the center of one of the most prominent band was calculated at around 3.5 Hz (Figure 8).

## 4 Discussion

We demonstrated a method to analyze the physiological brain pulsations in the human brain *in-vivo* using ultrafast 7 T EPI acquisitions, and an analytic approach to examine the dynamics of regional CSF signal pulsations. The raw visualization of the real-time signal (Figure 2) shows *in-vivo* CSF pulsations. The flow of CSF within the ventricles and in the subarachnoid space can be clearly visualized with changes in signal intensity. The time series and the frequency spectrum comparison between the collected physiological data and the EPI data (Figure 3) shows a direct alignment between the two types of data where the cardiac and respiration cycles can be observed in the EPI MRI data. The frequency analysis also shows consistent results across multiple volunteers, with similar frequency spectrums are observed.

Compared to previous studies (Fultz et al., 2019; Shanks et al., 2019; Yamada et al., 2020; Yang et al., 2022), we were able to achieve a greater frequency range, improved frequency resolution, and full brain coverage. This was made possible by utilizing ultrafast acquisition times (ranging from 51 to 155 ms), which allowed for whole-brain spectral analysis up to 9.8 Hz. Additionally, we employed high SNR and homogeneous images by using a 7 T MRI with a customized radiofrequency (RF) coil system (Santini et al., 2018; Krishnamurthy et al., 2019; Santini et al., 2021b). To optimize sequence parameters, we tailored the flip-angle to maximize the signal of the CSF flow and adjusted other parameters (e.g., acceleration factor) to minimize susceptibility-related distortions. We also selected a TE that could potentially capture the BOLD signal if functional connectivity data are warranted.

Based on the methodology implemented in this work, we can compare physiological brain pulsations across different tissue types and brain regions. In the frequency domain, there is evidence of more well-defined peaks in CSF regions than in other regions, with cGM following as the second most defined, and cWM as the third. This suggests a potential relationship between these measurements and CSF dynamics. Moreover, when comparing these peaks in terms of their respective magnitudes, areas under the curves, and bandwidths for a 3 dB drop, we observe low variability across different volunteers for similar segments, indicating potential biomarkers for brain pulsations analysis.

The creation of frequency masks allowed for an analysis of the spatial localization of each frequency band. The presence of heart rate frequencies (1.2 Hz) in the ventricles validates the analysis as the arterial pulse wave in the choroid plexus, for instance, is known to influence the CSF motion (Bilston et al., 2010; Martin et al., 2012; Iliff et al., 2013). Additionally, the presence of high frequencies (over 2 Hz) responses can suggest a more turbulent flow that also aligns with regions of the main cerebral aqueduct. This work provides a basis for identifying new biomarkers for brain fluid dynamics. For example, the frequency spectrum can be analyzed for different brain diseases. The lower frequency bands (below 1 Hz) contain physiological signals that corelate with the heart rate and breathing, so brain conditions that affect those variables can be analyzed directly from the MRI data. The magnitude of each band may also provide insights into the coupling between the heart and breathing rates with the CSF pulsations, which may correlate with clearance rate. On the other hand, the higher frequency bands (above 1.8 Hz), can be correlated with sleep cycles and potential sleep studies (Xie et al., 2013).

## 5 Conclusion

The development of non-invasive neuroimaging methods and biomarkers of brain fluid dynamics is essential for studying brain diseases. This work presents a novel methodology to characterize the frequency spectrum and spatial localization of CSF pulsations in the human brain. The use of ultrafast EPI in conjunction with 7 T human MRI and simultaneous collection of physiological data enabled the identification of primary components of CSF pulsations and their mapping spatially and temporally onto the MR image and physiological domains. The methodology showed low variability and repeatability *in-vivo*, making it a promising tool for potential studies of brain fluid dynamics and CSF flow. Future studies will explore this methodology in clinical studies to determine its implications for the diagnosis and treatment of brain diseases.

## Data availability statement

The raw data supporting the conclusions of this article will be made available by the authors, without undue reservation.

## Ethics statement

The studies involving humans were approved by University of Pittsburgh’s Institutional Review Board (identification number PRO17030036). The studies were conducted in accordance with the local legislation and institutional requirements. The participants provided their written informed consent to participate in this study.

## Author contributions

TM: Conceptualization, Formal analysis, Investigation, Methodology, Software, Writing – original draft, Writing – review & editing. BD: Investigation, Methodology, Software, Writing – review & editing, Formal analysis, Visualization. MW: Conceptualization, Methodology, Software, Writing – original draft, Writing – review & editing. KW: Conceptualization, Writing – original draft, Writing – review & editing. DM: Writing – original draft, Writing – review & editing, Conceptualization. JI: Conceptualization, Methodology, Investigation, Writing – original draft, Writing – review & editing, Resources. HA: Conceptualization, Funding acquisition, Writing – original draft, Writing – review & editing. TS: Formal analysis, Supervision, Writing – original draft, Writing – review & editing, Conceptualization, Investigation, Methodology, Software. TI: Conceptualization, Funding acquisition, Investigation, Methodology, Resources, Supervision, Writing – original draft, Writing – review & editing.
