# Supplementary material for: Characterization of pulsations in the brain and cerebrospinal fluid using ultra-high field magnetic resonance imaging
Source: Front Neurosci. 2024 May 9;18:1305939. doi: 10.3389/fnins.2024.1305939 (PMC11112101; doi:10.3389/fnins.2024.1305939)
Supplement: Supplementary file 1 [file Image_1.pdf]

Supplementary Material

Phantom FFT Analysis Overview

In our discussion on the development of an algorithm for identifying FFT peaks (Section 2.4.1 Spectral Peaks Identification), we underscored the method of obtaining a lower envelope across all spectra for normalization purposes. This method was inspired by observations from conducting similar fast EPI sequences on a phantom, with sequence parameters as detailed in Section 2.1 (Image Acquisition).

Figure s1 demonstrates the FFT results obtained from performing an analogous EPI sequence on a phantom. A notable frequency-dependent amplitude decrease is evident. Considering that the FFT from each brain region presents distinct frequency-dependent characteristics, as depicted in Figure 5a, it becomes clear that creating a universal curve from phantom data, applicable to all regions, is not feasible. As a result, we opted to emulate this pattern by accumulating the minimum values along the frequency axis. This approach allows us to adapt our analysis to the variability observed across different brain regions and among participants.

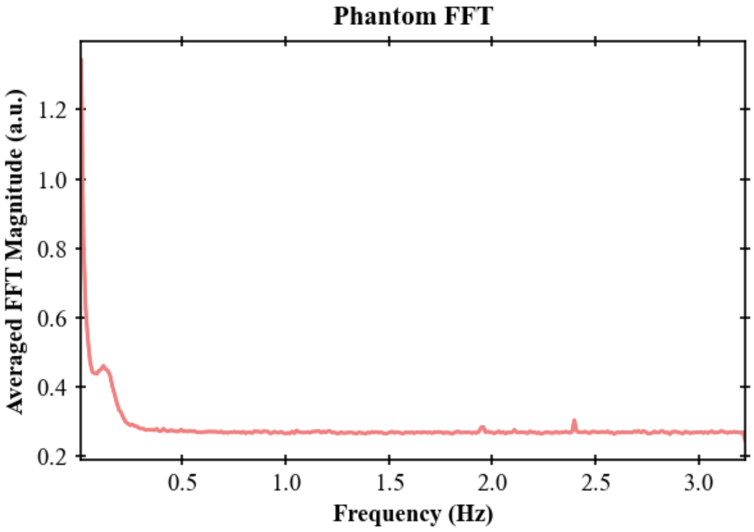

Figure s1: FFT spectrum obtained from the fast EPI sequence run on a phantom.
